# Health informatics publication trends in Saudi Arabia: a bibliometric analysis over the last twenty-four years

**DOI:** 10.5195/jmla.2021.1072

**Published:** 2021-04-01

**Authors:** Samar Binkheder, Raniah Aldekhyyel, Jwaher Almulhem

**Affiliations:** 1 sbinkheder@ksu.edu.sa, Assistant Professor of Biomedical and Health Informatics, College of Medicine, King Saud University, Riyadh, Kingdom of Saudi Arabia; 2 raldekhyyel@ksu.edu.sa, Assistant Professor of Biomedical and Health Informatics, College of Medicine, King Saud University, Riyadh, Kingdom of Saudi Arabia; 3 jalmulhem@ksu.edu.sa, Assistant Professor of Biomedical and Health Informatics, College of Medicine, King Saud University, Riyadh, Kingdom of Saudi Arabia

**Keywords:** biomedical informatics, health informatics, clinical informatics, consumer health informatics, public health informatics, bibliometric analysis

## Abstract

**Objective:**

Understanding health informatics (HI) publication trends in Saudi Arabia may serve as a framework for future research efforts and contribute toward meeting national “e-Health” goals. The authors’ intention was to understand the state of the HI field in Saudi Arabia by exploring publication trends and their alignment with national goals.

**Methods:**

A scoping review was performed to identify HI publications from Saudi Arabia in PubMed, Embase, and Web of Science. We analyzed publication trends based on topics, keywords, and how they align with the Ministry of Health's (MOH's) “digital health journey” framework.

**Results:**

The total number of publications included was 242. We found 1 (0.4%) publication in 1995–1999, 11 (4.5%) publications in 2000–2009, and 230 (95.0%) publications in 2010–2019. We categorized publications into 3 main HI fields and 4 subfields: 73.1% (n=177) of publications were in clinical informatics (85.1%, n=151 medical informatics; 5.6%, n=10 pharmacy informatics; 6.8%, n=12 nursing informatics; 2.3%, n=4 dental informatics); 22.3% (n=54) were in consumer health informatics; and 4.5% (n=11) were in public health informatics. The most common keyword was “medical informatics” (21.5%, n=52). MOH framework–based analysis showed that most publications were categorized as “digitally enabled care” and “digital health foundations.”

**Conclusions:**

The years of 2000–2009 may be seen as an infancy stage of the HI field in Saudi Arabia. Exploring how the Saudi Arabian MOH's e-Health initiatives may influence research is valuable for advancing the field. Data exchange and interoperability, artificial intelligence, and intelligent health enterprises might be future research directions in Saudi Arabia.

## INTRODUCTION

Biomedical informatics (BMI) is defined as “the interdisciplinary field that studies and pursues the effective uses of biomedical data, information, and knowledge for scientific inquiry, problem-solving, and decision making, motivated by efforts to improve human health” [1]. BMI is a fast-evolving field and the core scientific discipline supporting both applied research and practice, which includes health informatics (HI) and subfields [1]. Its interdisciplinary nature and its relevance to health care advancement are major contributing factors [2, 3].

Literature trends and bibliometric analysis of published research help quantify insights into the current and future trends of the field, research efforts, and educational programs development [4–6]. During the past five years, research efforts examining publication trends in the HI field show great attention to the areas of clinical informatics, consumer health informatics, and mobile health [7, 8]. This focus may be due to the increased use of smartphones and other technologies [7] and is expected to continue growing in the future [8]. In addition, many researchers have explored how specific policies and regulations may affect the advancement of the field. For example, in the United States, key findings of the American Medical Informatics Association's (AMIA's) review on clinical and consumer informatics topics show that newly established US policies for electronic health record (EHR) implementation and evaluation introduce new challenges in health care, such as data interoperability, the impact of decision support systems, predictive models and their utilization, mobile applications and EHR systems integration, and the early stages of interactive natural language systems development [9].

In Saudi Arabia, the first institution obtained access to the Internet in 1993 [10]. At that time, the national health reform committee identified a lack of HI applications and information systems as a challenge within the health sector. Accordingly, a task force was developed in 2002 to build a national EHR and to expand electronic health services, including telemedicine. As a result, the Saudi Association for Health Informatics [11], the first official HI association in the country, was established in 2005 [12], and the Ministry of Health (MOH), guided by the country's 2030 vision, launched several initiatives in 2010 to support the development of a national “e-Health” strategy, which included a ten-year roadmap based on patient-centric care [13].

The MOH positions e-Health as the primary transformative and enabler agent, with the primary goal of the e-Health strategy being to provide care for patients, connect providers, measure performance, and transform health care delivery to standardized care [13]. Guiding and supporting research was specifically stated as one of the e-Health objectives [14], with the aim of improving health care through utilization of information technology and digital transformation [15]. The MOH also developed a “digital health strategy” highlighting the need for rapid digital change and reinvention [14]. Examples of projects that have been initialized or completed as part of the e-Health initiative are a medical records improvement program, referral system (Ehalty), unified portal of health services, health electronic surveillance network, poison control e-system (Awtar), neonatal protection system, hospitals’ serious incidents registration e-system, and premarital screening system [16, 17]. These national efforts and the MOH's e-Health initiative have played a big role in the evolution of the HI field in Saudi Arabia during the last decade.

Understanding current HI publication trends in Saudi Arabia may contribute to meeting national e-Health goals. Publications in scientific journals offer insights into topics and trends in HI research [2, 3, 18] and can identify gaps in research that support the advancement of HI [3]. To the best of our knowledge, no studies have explored HI research trends, particularly in Saudi Arabia. As the role of governing policies on the future of HI requires exploration through published literature and open discussions by experts in the field of HI [9], the authors aimed to explore trends in HI research in Saudi Arabia and understand how these publications might be aligned with the MOH's digital health plans. Ultimately, we intend to understand the past, current, and future state of the HI field, which includes clinical informatics, consumer health informatics, and public health informatics.

## METHODS

### The Ministry of Health's (MOH's) “e-Health” strategy overview

In 2010, the MOH initiated the 2010–2020 roadmap for the national e-Health strategy, separated into two five-year phases, which was launched in early 2011 [13, 14]. The evolution of digital health first started in 2010 with some standalone systems that had limited functionalities and lacked interoperability [14]. The MOH's objective for the national e-Health system is to improve individuals’ personal experiences, increase efficiency and performance, improve health outcomes and equity, enable health providers to deliver better services, and provide evidence for policy, research, and planning [13, 14]. To measure the country's digital capabilities as part of the national e-Health strategy, the MOH developed a framework called the “digital health journey,” which consists of six levels: (1) digital health foundations; (2) digitally enabled care (e.g., EHRs and decision support); (3) smart care (e.g., precision medicine, artificial intelligence, robotics, and medical printing); (4) care anywhere (e.g., virtual care, connected care teams, and connected homes); (5) empowered care (e.g., models of care, patient experience, and personal health data); and (6) intelligent health enterprises (e.g., seamless financing; data-driven, value-based, accountable care; and end-to-end systems) [14]. We used the six levels in this “digital health journey” as a framework for our study to categorize HI publication trends.

### Search strategy

We conducted a scoping review to identify publications within the field of HI using three databases: PubMed, Embase, and Web of Science (WOS). A librarian, who is an expert researcher in the field, was consulted for search keywords and database selection. The search queries for each HI discipline (supplemental Appendix A) were based on the AMIA Board white paper for defining the BMI field [1]. All search queries were accompanied by “Saudi Arabia” or “Saudi” to limit our results to publications written by authors affiliated with Saudi institutions. We included all publications until December 31, 2019.

### Screening and study selection

**Figure 1:**
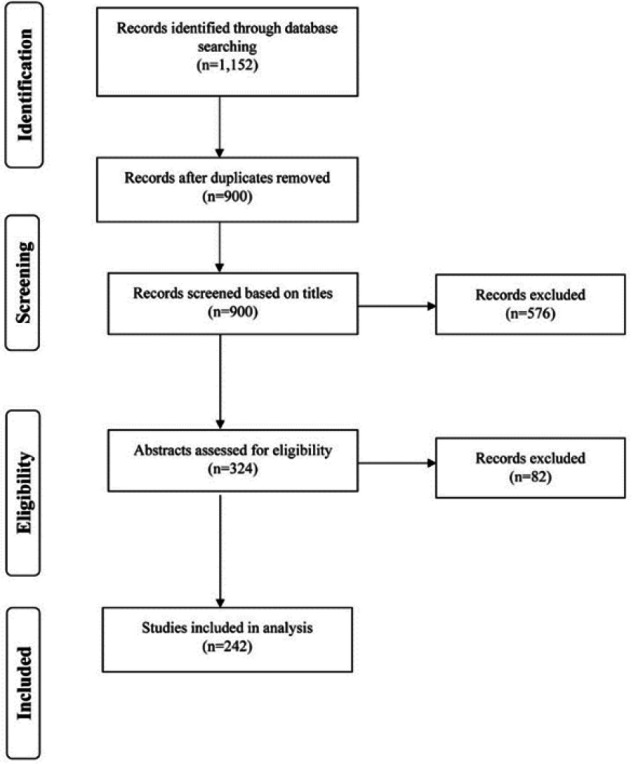
Preferred Reporting Items for Systematic Reviews and Meta-Analysis (PRISMA) diagram

Figure 1 shows our search and screening process. Database searching yielded a total of 1,152 records. After duplicate records were removed, a total of 900 records were screened. Three BMI experts performed the title and abstract screening using Rayyan, a web application that facilitates record screening for systematic reviews [19]. The records were divided into three subsets, with each subset assigned to two reviewers for independent screening. Discrepancies were resolved by the third reviewer. The inclusion criteria were (1) first author from or study location in Saudi Arabia and (2) an HI-related topic. The exclusion criteria were (1) records without abstracts, (2) proposals, (3) abstract language not Arabic or English, and (4) non-HI topics, including bioinformatics, structural (i.e., imaging) informatics, and informatics in translational science (i.e., translational bioinformatics and clinical research informatics). Finally, records that were not accessible by our institutional access or the Saudi Digital Library were excluded. After removing a total of 658 records based on title and abstract screening, we included a final set of 242 records.

### Data extraction and analysis

Each publication's metadata were downloaded from PubMed, Embase, and WOS databases, which included abstract, publication year, journal name, and keywords (Medical Subject Headings [MeSH] from PubMed, Emtree from Embase, and authors’ keywords from WOS). We created a data extraction form using Google forms [20] for further analysis, which included the institutions/affiliations of all authors; whether first authors had Saudi affiliations; study location; data source (i.e., patients or medical data such as EHR data, surveys and/or questionnaires, interviews or focus groups, patient or disease registries, clinical or health care research datasets, and other data [e.g., social media]); publication type (e.g., research and applications, case reports, review, and other); type of methodology (i.e., qualitative, quantitative, mixed review, and other); and source of publication (i.e., journal, proceeding, and other) (supplemental Appendix B.).

Using titles and abstracts, we assigned HI fields and subfields to each publication, which consisted of clinical informatics (medical informatics, nursing informatics, pharmacy informatics, and dental informatics), public health informatics, and consumer health informatics. Publications were then categorized based on the MOH's “digital health journey” framework [14]. Again, the set of records was divided into three subsets, with each subset assigned to two reviewers for independent categorization. Discrepancies were resolved by a third reviewer. We also performed descriptive analysis to identify trends in HI in Saudi Arabia between 1995 and 2019. We used Microsoft Excel and Tableau [21] for data analysis and visualization.

## RESULTS

Analyses were based on 3 time periods: the first period was a 4-year interval from 1995 to 1999, and the subsequent 2 periods were 10-year intervals from 2000–2009 and 2010–2019. A total of 242 publications were included in our study. We found only 1 (0.4%) publication from 1995 to 1999, 11 (4.5%) from 2000–2009, and 230 (95%) from 2010–2019.

### Publication-based analysis

Table 1 provides a descriptive summary of the included publications. There were 3 publication sources: 60.7% (n=147) journals, 38.8% (n=94) proceedings, and 0.4% (n=1) books. The most common publication type (74%, n=179) was “research and applications.” The study location was mostly in Saudi Arabia (57.5%, n=140). Other study locations included the United States (1.9%, n=3) [22–24], Malaysia (1.9%, n=3) [25–27], and Canada (0.8%, n=2) [28, 29]. The majority of publications (35.1%, n=85) used quantitative methods, and the minority of publications (9.1%, n=22) used mixed methods.

**Table 1:** A descriptive summary of health informatics (HI) publications by Saudi-affiliated authors

Source of publication	Publication type	Study location	Type of methodology										Total	
			Quantitative		Qualitative		Mixed		Review		Other			
Journal	Research and applications	Saudi Arabia	43	(17.77%)	5	(2.07%)	8	(3.31%)	2	(0.83%)	14	(5.79%)	118	(48.76%)
		Malaysia	1	(0.41%)	1	(0.41%)	—		—		—			
		Not specified	5	(2.07%)	3	(1.24%)	1	(0.41%)	—		20	(8.26%)		
		Canada	1	(0.41%)	1	(0.41%)	—		—		—			
		Ethiopia	1	(0.41%)	—		—		—		—			
		Iraq	1	(0.41%)	—		—		—		—			
		Jordan	1	(0.41%)	—		—		—		—			
		Madagascar	1	(0.41%)	—		—		—		—			
		United States	1	(0.41%)	—		1	(0.41%)	—		1	(0.41%)		
		Multiple countries	5	(2.07%)	—		1	(0.41%)	—		—			
	Review	Saudi Arabia	—		—		—		6	(2.48%)	—		19	(7.85%)
		Not specified	—		—		—		13	(5.37%)	—			
	Case reports	Saudi Arabia	2	(0.83%)	—		—		2	(0.83%)	—			
	Brief communication	Multiple countries	1	(0.41%)	—		—		1	(0.41%)	—			
	Commentary	Saudi Arabia	—		—		1	(0.41%)	2	(0.83%)	—			
		Not specified	—		—		1	(0.41%)	—		—			
	Correspondence	China	—		—		1	(0.41%)	1	(0.41%)	—			
	Editorial	Not specified	—		—		2	(0.83%)	2	(0.83%)	—			
	Perspective	Saudi Arabia	—		—		1	(0.41%)	1	(0.41%)	—			
	Report	Saudi Arabia	—		—		1	(0.41%)	1	(0.41%)	—			
Proceeding	Research and applications	Saudi Arabia	12	(4.96%)	9	(3.72%)	7	(2.89%)	1	(0.41%)	9	(3.72%)	61	(25.21%)
		Not specified	7	(2.89%)	2	(0.83%)	—		1	(0.41%)	10	(4.13%)		
		Multiple countries	2	(0.83%)	—		—		—		—			
		United Kingdom	—		1	(0.41%)	—		—		—			
	Review	Saudi Arabia	—		2	(0.83%)	4	(1.65%)	—		—		12	(4.96%)
		Not specified	—		—		6	(2.48%)	—		—			
	Case reports	Saudi Arabia	1	(0.41%)	4	(1.65%)	1	(0.41%)	1	(0.41%)	5	(2.07%)	15	(6.20%)
		Malaysia	—		1	(0.41%)	—		—		—			
		Not specified	—		—		2	(0.83%)	—		—			
	Perspective	Saudi Arabia	—		—		1	(0.41%)	6	(2.48%)	—			
		Not specified	—		—		5	(2.07%)			—			
Book	Chapter	Not specified	—		—		1	(0.41%)	1	(0.41%)	—			
Total			85	(35.12%)	26	(10.74%)	22	(9.09%)	34	(14.05%)	75	(30.99%)	242	(100.00%)

**Figure 2:**
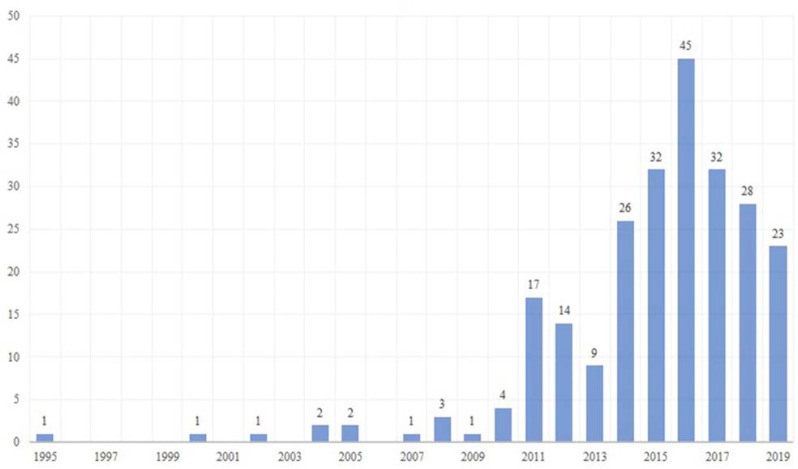
Growth in the number of publications over years

The first publication found was in 1995 (Figure 2). We observed a continuous increase in the number of publications from 2010–2016, when the highest peak (n=45, 18.6%) occurred. However, there was a decrease in the number of publications in 2017, 2018, and 2019. We found authors with Saudi affiliations as first authors in 203 (83.9%) publications. When we investigated the top institutions and cities for authors with Saudi affiliations (supplemental Appendix C), the institution with the most publications was King Saud bin Abdulaziz University for Health Sciences with 105 (43.4%) publications. The city of Riyadh had the highest number of contributing institutions’ publications.

We highlighted some major data sources that were used in HI publications. For publications in 1995–1999, there were no data sources found. For publications in 2000–2009, data sources used were surveys or questionnaire data [30], patient or medical data [31–33], clinical or health care research datasets [34], and patient or disease registries [35]. For publications in 2010–2019, more data sources were used, including surveys or questionnaire data [12, 24, 26, 36–96], interviews or focus groups [25, 27, 28, 37, 69, 94, 97–119], patient or medical data [23, 120–142], clinical or health care research datasets [23, 143–153], patient or disease registries [29, 67, 154, 155], and social media (Facebook [156, 157], Twitter [158, 159], Quora [22], and WhatsApp [160]) and new social media datasets [161].

### Topic-based analysis

**Figure 3:**
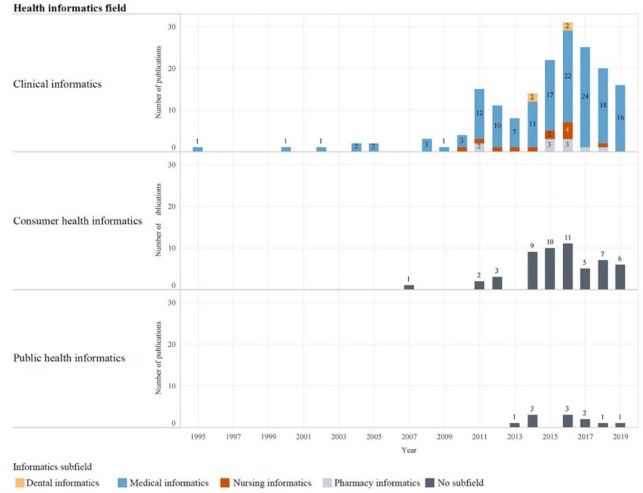
Topics of health informatics publications over time

We investigated trends in research topics in publications based on HI fields and subfields (Figure 3). For 1995–1999, the first publication was in clinical informatics (subfield: medical informatics). For 2000–2009, all publications were in clinical informatics (subfield: medical informatics) except for 2007, when publications for consumer health informatics first appeared. For 2010–2019, there were new emerging trends in all HI fields and subfields within clinical informatics. Additionally, publications in public health informatics first appeared in 2013. Over the years, publication topics were mostly related to clinical informatics (73.1%, n=177)—including the subfields of medical informatics (85.3%, n=151), pharmacy informatics (5.6%, n=10), nursing informatics (6.8%, n=12), and dental informatics (2.3%, n=4)—with fewer publications related to consumer health informatics (22.3%, n=54) and public health informatics (4.5%, n=11).

### Keyword-based analysis

To conduct keyword-based analysis, we extracted keywords from PubMed (MeSH keywords), Embase (Emtree keywords), and WOS (authors’ keywords). Non-HI related keywords were removed. The only publication in 1995–1999 [162] was associated with the keywords “medical informatics,” “hospital information systems,” and “information science.” For 2000–2009, new HI keywords emerged, including “electronic health record”; “Internet”; “doctor patient relationship”; “medical information”; “computer security”; “database management systems”; “registries”; “decision support systems, management”; “medical records systems, computerized”; “picture archiving and communication system”; “telemedicine”; “reminder systems”; “computer assisted diagnosis”; “prediction”; and “algorithm.” For 2010–2019, keywords emerged with new HI fields, such as “health informatics,” “public health informatics,” “dental informatics,” “nursing informatics,” and “consumer health informatics.” There was a trend in patient-oriented keywords, such as “patient compliance,” “patient safety,” “patient care management,” “patient education as topic,” “patient satisfaction,” and “e-patients.” Keywords emerged in HI subdomains, such as “medical order entry systems,” “health information exchange,” “software,” “clinical pharmacy information systems,” “human computer interaction,” “sensitive health information,” and “systems integration.” Furthermore, keywords in data sciences, analytics, and technologies appeared, such as “data mining,” “data analysis,” “medical record linkage,” “mobile application,” “social media,” “ontology development,” “machine learning,” “big data,” “facial recognition,” “semantics,” “named entity recognition,” and “cloud computing.” Many of these keywords occurred only once in publications.

For the most frequent HI-relevant keywords in publications, Table 2 shows the top 30 keywords. “Medical informatics” was the earliest and most frequently occurring keyword (21.5%, n=52), appearing in every year except for 2002, 2007, and 2009. The “Internet” keyword emerged in year 2002. “Health informatics” first emerged in 2011 and then appeared yearly after 2013. The first occurrence of “social media” was in 2015. “Electronic medical record” and “telemedicine” first emerged in 2008. Considering these top 30 keywords, 2016 was the year with the highest frequency of HI keywords (25.2%, n=61).

**Table 2:** Top 30 keywords in publications

	Year																	
Keyword	1995	2002	2004	2005	2007	2008	2009	2010	2011	2012	2013	2014	2015	2016	2017	2018	2019	Grand total
Medical informatics	1		1	2		1		1	3	2	2	6	6	9	6	3	9	52
Internet		1		1		1		1	4	2	1		4	2	2	1		20
Health informatics									1		1	3	2	3	4	2	4	20
Social media													4	6	5	2	2	19
Electronic medical record						2				2		4		2	2	4	2	18
Telemedicine						1						2	4	2	3	2	3	17
Medical order entry systems									1	2	3	1	2	2	1	1		13
Health care quality						1							1	3	4	2	2	13
Electronic health records							1			2	1	4		3	1		1	13
Electronic health record									1					3	4	2	3	13
Medical information system						1			1	2		2	1	2		1	2	12
Medical information				2						1				3	3	2	1	12
Privacy									1			1	1		3	1	4	11
Information processing									1	1		1		2	4	1	1	11
Technology									1			1	1	2	1	2	2	10
Information technology									1				1	1	1	4	2	10
Workflow											4	1	2	1		1		9
Patient safety							1				1	1	1	2	1	1	1	9
Health care delivery						1							2	2	3		1	9
Data mining								2		1		1		1	2	1	1	9
Smartphone												1	1	3	1	2		8
Knowledge									2			1	1	2	1		1	8
Health information systems												5	1		2			8
Content analysis									2	1			1	1	2		1	8
Systems integration										2	1	1	3					7
Software									1			3	2	1				7
Mobile application													2	3	1	1		7
Medical record linkage								1		1	1	2	2					7
Machine learning															1	1	5	7
Information systems								1	1		1		3			1		7
Grand total	1	1	1	5	0	8	2	6	21	19	16	41	48	61	58	38	48	374

Light blue=lowest frequency, darkest blue=highest frequency.

### “Digital health journal” framework-based analysis

We categorized HI publications based on the MOH's digital health journey framework. Only 1 publication was categorized under the first level “digital health foundations” in 1995–1999. Publications in 2000–2009 were categorized as 1.7% (n=4) “digital health foundations,” 1.7% (n=4) “digitally enabled care,” 0.8% (n=2) “smart care,” and 0.4% (n=1) “care anywhere.” Publications in 2010–2019 were categorized as 27.7% (n=67) “digitally enabled care,” 24.0% (n=58) “digital health foundations,” 17.4% (n=42) “smart care,” 17.4% (n=42) “empowered care,” 6.2% (n=15) “care anywhere,” and 2.5% (n=6) “intelligent health enterprises.”

**Figure 4:**
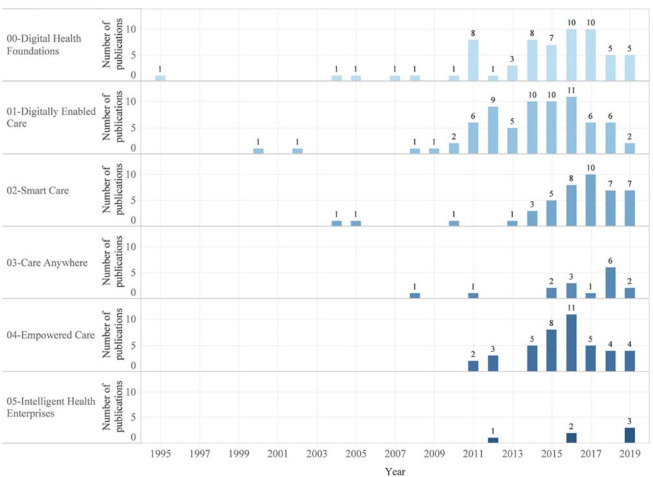
Trends in publications categorized based on the Ministry of Health's (MOH's) “digital health journey” framework over time

Most publications were categorized as “digitally enabled care” (29.3%, n=71) or “digital health foundations” (26.0%, n=63) (Figure 4). Few publications (2.5%, n=6) were categorized as “intelligent health enterprises.” “Digital health foundations” showed a continuous upward trend in published research (except for between 2000 and 2002) starting in 1995, with the first publication. Publications related to “digital health foundations” reached peaks (4.1%, n=10) in 2016 and 2017. Publications related to “digitally enabled care” started to appear in 2000 and reached a peak (4.5%, n=11) in 2016. Additionally, publications categorized as “smart care” started to appear in 2004 and reached a peak (4.1%, n=10) in 2017; those categorized as “care anywhere” started to appear in 2008 and reached a peak (2.5%, n=6) in 2018; and those categorized as “empowered care” started to appear in 2011 and reached a peak in (4.5%, n=11) in 2016. Lastly, publications categorized as “intelligent health enterprises” appeared only in 3 years: 2012, 2016, and 2019.

## DISCUSSION

We analyzed trends in HI publications by Saudi-affiliated authors over the past two decades. In 1995–1999, there was only one publication [162], which was published before the health care services review conducted by the health reform committee in 2000 [12]. This was the first HI publication with a special focus on hospital information systems. This publication was categorized under the first level of the MOH's digital framework, indicating the emergence of the HI field in Saudi Arabia as early as 1995.

In 2000–2009, there was an increase in the number of publications. Data sources varied during this period but still were limited, with medical and consumer informatics topics being top trends. The keyword “Internet” first appeared during this period, which might be due to the increased use of the Internet in Saudi Arabia at the same time [10, 163]. King Saud bin Abdulaziz University for Health Sciences’ establishment of the Saudi Association for Health Informatics [11] and the HI master program in 2005 [12] may have contributed to the highest number of publications and the occurrence of more specialized HI keywords, such “electronic medical record” and “telemedicine.” Other keywords emerged in one publication (e.g., “prediction” and “algorithm”) [35], which aligned with “smart care” in our framework-based analysis. This time period has been seen as the maturity period of medical informatics [164]; however, HI publications in 2000–2009 were mainly aligned with the MOH's first two levels, which may indicate that this period was an infancy stage in Saudi Arabia.

The highest number of publications was seen in 2010–2019. We believe the rise in the number of publications starting in 2010 may have been stimulated by the MOH initiative and e-Health objectives for health transformation as part of the Saudi 2030 vision. During this time period, there was a new trend with a few publications that used social media as a data source, which also emerged in the keyword analysis. Topic-based and keyword-based analyses showed increasing trends in clinical informatics and consumer health informatics and new trends in public health informatics and clinical informatics subfields. Moreover, there were trends in patient-oriented keywords. These trends were consistent with those found in previous studies [7, 8, 164]. Furthermore, there was an emergence of data science and analytics subdomains seen in keywords, such as “machine learning” [29, 96, 124, 130, 140, 165, 166], “data mining” [36, 52, 123, 134, 140, 143, 167–169], and “big data” [170–172], which was also reported by another study during the same time period [164]. Our framework-based analysis showed a distribution of publications across all levels, providing evidence of huge progress and variation in research efforts in comparison with the two previous time periods.

Our results provide several insights into current and future HI trends in Saudi Arabia. First, we found that the use of multiple sources of data for research in Saudi Arabia, such as patient or medical and real-time data, is still limited. For example, we found that most publications in our study used questionnaires, surveys, or interviews as data sources, which might pose some limitations, including limited reliability [173] and unrepresentative samples. For a fast-evolving field concerned with data science and big data, reliance on limited data sources is not sufficient to advance the HI field, which requires utilizing a variety of informatics platforms and data [173, 174]. Therefore, we believe that there is a need to not only collect health care data, but also understand and analyze data and utilize advanced technologies to derive data-driven decisions. Limited use of data sources might be due to a lack of clear regulations for data governance, including sharing sensitive data and repositories, which might limit the secondary use of health data.

With the increasing complexity of the health care sectors and fragmentation of digital services, the digital health vision was established to address such issues [14]. Recently, the Saudi Data & Artificial Intelligence Authority was established in 2019 [173, 175], and we believe that this will largely contribute to data governance regulations in Saudi Arabia. Second, unlike trends reported in the AMIA review [9], we found only one publication on data interoperability [119] and no research trends in some subdomains, such as natural language systems. Even though “health information exchange” appeared in our keyword-based analysis, keywords on standard systems for messaging and terminologies were not found. With the absence of systems that support interoperability and health information exchange, transferring patients’ medical records between different Saudi health care organizations remains a challenge due to the varying number of governing health care bodies [176]. The national strategy highlights the importance of standardization of information and processes and data completeness, which are important components that enable health information exchange and interoperability and contribute to advancing data analytics and research [13].

Third, even though the e-Health initiative is led by the MOH, we found a low number of MOH publications. Additionally, although we expected a growth of publications over years, we observed a decrease in the number of publications after 2016, which may reflect a lack of research efforts, funding sources, data sharing, and research centers. We believe that more research investment [177] and funding programs are needed, which can offer an opportunity to accelerate and increase HI publications in Saudi Arabia. Fourth, similar to previous studies that show many biomedical publications from Riyadh [177, 178], we also found that most HI publications were from Riyadh. This might be because Riyadh is the capital city, where most funding agencies are located. Lastly, we expect increases in publications in 2020–2030 and on the topics of data exchange and interoperability, artificial intelligence, national EHR, and intelligent health enterprises.

There are some limitations in our study. Our keyword-based analysis might have some limitations due to the use of different terminology sources (MeSH, Emtree, and WOS author keywords) in which some keywords might be semantically or syntactically equivalent. Matching similar keywords requires text mining and similarity-based methods that were out of the scope of this study. As this study is a scoping review, it might not include all HI-related publications due to the multidisciplinary nature and broad nature of the field [164, 179]. Specifically, we acknowledge that for this study, our selection of keywords in search queries was based on major AMIA classification and did not focus on subdomains. Future studies could use a more comprehensive search strategy to include more HI keywords and subdomains. Additionally, if Saudi authors did not specify their affiliations or populations of study (e.g., Saudi students studying abroad), our search strategy would not have captured these publications. Finally, we examined the publications only quantitatively and not qualitatively. Future work could qualitatively evaluate HI publications in Saudi Arabia.

## CONCLUSIONS

Based on published research, 2000–2009 may be seen as the infancy stage of the HI field in Saudi Arabia. The highest number of HI publications was during the years 2010–2019. However, the generally low number of publications may reflect a lack of research efforts, funding sources, data sharing, and research centers. Due to the intradisciplinary nature of HI, we believe that exploring research publication trends and understanding how Saudi's initiatives and governing bodies may have an effect on research is valuable to the advancement of the discipline. This is especially true given variations in policies and regulations across countries. More HI publications that focus on data exchange and interoperability, artificial intelligence, national EHR, and intelligent health enterprises might be future directions in Saudi Arabia, in alignment with the MOH's digital health journey framework. Finally, there is a need to increase funding opportunities, facilitate data sharing, understand and analyze health care data, and utilize advanced technologies to derive data-driven decisions.

## Data Availability

Data collected by authors is available through an Open Science Framework (OSF) project page at https://osf.io/q4ev3/?view_only=7d47b0bbfcdb43f19fad71bbb04f44de. Raw data for publications can be downloaded directly from PubMed, Embase, and Web of Science.

## SUPPLEMENTAL FILES

**Appendix A:** Keywords search queries

**Appendix B:** Google form for data extraction

**Appendix C:** (a) Top ten Saudi institutions affiliations for all authors, and (b) the geographical distribution of publications in Saudi's cities based on author affiliation

## References

[1] Kulikowski CA, Shortliffe EH, Currie LM, Elkin PL, Hunter LE, Johnson TR, Kalet IJ, Lenert LA, Musen MA, Ozbolt JG, Smith JW, Tarczy-Hornoch PZ, Williamson JJ. AMIA board white paper: definition of biomedical informatics and specification of core competencies for graduate education in the discipline. J Am Med Inform Assoc. 2012 Nov-Dec;19(6):931–8. DOI: 10.1136/amiajnl-2012-001053. 22683918 PMC3534470

[2] Kim HE, Jiang X, Kim J, Ohno-Machado L. Trends in biomedical informatics: most cited topics from recent years. J Am Med Inform Assoc. 2011 12;18(suppl 1):i166–70. DOI: 10.1136/amiajnl-2011-000706. 22180873 PMC3241182

[3] Kim H, Ohno-Machado L, Oh J, Jiang X. Trends in publication of nursing informatics research. AMIA Annu Symp Proc. 2014 2014:805–14. 25954387 PMC4419947

[4] Koch S. Current trends in biomedical and health informatics. Methods Inf Med. 2019 9;58(02–03):61–2. DOI: 10.1055/s-0039-1695764. 31514206

[5] Wang L, Topaz M, Plasek JM, Zhou L. Content and trends in medical informatics publications over the past two decades. Stud Health Technol Inform. 2017 245:968–72. 29295244

[6] Mendis K. Health informatics research in Australia: retrospective analysis using PubMed. J Innov Health Inform. 2007:7. DOI: 10.14236/jhi.v15i1.641. 17612471

[7] Lai AM, Hsueh PS, Choi YK, Austin RR. Present and future trends in consumer health informatics and patient-generated health data. Yearb Med Inform. 2017 8;26(1):152–9. DOI: 10.15265/IY-2017-016. 29063559 PMC6239232

[8] Gulkesen KH, Haux R. Research subjects and research trends in medical informatics. Methods Inf Med. 2019 6;58(S 01):e1–13. DOI: 10.1055/s-0039-1681107. 30919405

[9] Roberts K, Boland MR, Pruinelli L, Dcruz J, Berry A, Georgsson M, Hazen R, Sarmiento RF, Backonja U, Yu KH, Jiang Y, Brennan PF. Biomedical informatics advancing the national health agenda: the AMIA 2015 year-in-review in clinical and consumer informatics. J Am Med Inform Assoc. 2017 4 1;24(e1):e185–90. DOI: 10.1093/jamia/ocw103. 27497798 PMC6080724

[10] Alshahrani A, Stewart D, MacLure K. A systematic review of the adoption and acceptance of ehealth in Saudi Arabia: views of multiple stakeholders. Int J Med Inform. 2019 8;128:7–17. DOI: 10.1016/j.ijmedinf.2019.05.007. 31160014

[11] Saudi Association for Health Informatics. SAHI [Internet]. The Association [cited 29 Jan 2021]. <http://www.sahi.org.sa>.

[12] Altuwaijri MM. Supporting the Saudi e-health initiative: the master of health informatics programme at KSAU-HS. East Mediterr Health J. 2010 1;16(1):119–24. 20214169

[13] Ministry of Health. Ministry vision “e-Health” [Internet]. The Ministry [cited May 2020]. <https://www.moh.gov.sa/en/Ministry/nehs/Pages/default.aspx>.

[14] Ministry of Health. Digital health strategy update 2018 [Internet]. The Ministry; 2018 [cited May 2020]. <https://www.moh.gov.sa/Ministry/vro/eHealth/Documents/MoH-Digital-Health-Strategy-Update.pdf>.

[15] Alharbi MF. An analysis of the Saudi health-care system's readiness to change in the context of the Saudi national health-care plan in vision 2030. Int J Health Sci (Qassim). 2018 May-Jun;12(3):83–7. PMC5969787 29896076

[16] Ministry of Health. MOH initiatives & projects: MOH initiatives [Internet]. The Ministry [cited Sep 2020]. <https://www.moh.gov.sa/en/Ministry/Projects/Pages/pre.aspx>.

[17] Ministry of Health. MOH initiatives & projects: accomplished MOH projects [Internet]. The Ministry [cited Sep 2020]. <https://www.moh.gov.sa/en/Ministry/Projects/Pages/DoneProjects.aspx>.

[18] Jiang X, Tse K, Wang S, Doan S, Kim H, Ohno-Machado L. Recent trends in biomedical informatics: a study based on JAMIA articles. J Am Med Inform Assoc. 2013 12;20(e2):e198–205. DOI: 10.1136/amiajnl-2013-002429. 24214018 PMC3861936

[19] Ouzzani M, Hammady H, Fedorowicz Z, Elmagarmid A. Rayyan—a web and mobile app for systematic reviews. Syst Rev. 2016 12 5;5(1):210. DOI: 10.1186/s13643-016-0384-4. 27919275 PMC5139140

[20] Google. Google forms [Internet]. Google [cited Mar 2020]. <https://www.google.com/forms/about/>.

[21] Tableau [Internet]. Tableau Software [cited May 2020]. <https://www.tableau.com/products/desktop>.

[22] Alasmari A, Zhou L. How multimorbid health information consumers interact in an online community Q&A platform. Int J Med Inform. 2019 11;131:103958. DOI: 10.1016/j.ijmedinf.2019.103958. 31521012

[23] Aldekhyyel RN, Melton GB, Lindgren B, Wang Y, Pitt MB. Linking pediatrics patients and nurses with the pharmacy and electronic health record system through the inpatient television: a novel interactive pain-management tool. Hosp Pediatr. 2018 9;8(9):588–92. DOI: 10.1542/hpeds.2018-0096. 30115680 PMC6226310

[24] Rajamani S, Chen ES, Lindemann E, Aldekhyyel R, Wang Y, Melton GB. Representation of occupational information across resources and validation of the occupational data for health model. J Am Med Inform Assoc. 2018 2;25(2):197–205. DOI: 10.1093/jamia/ocx035. 28444213 PMC6080809

[25] Zakaria N, Ramli R. Physical factors that influence patients’ privacy perception toward a psychiatric behavioral monitoring system: a qualitative study. Neuropsychiatr Dis Treat. 2018 12 29;14:117–28. DOI: 10.2147/NDT.S115261. 29343963 PMC5751803

[26] Mohd Salleh MI, Zakaria N, Abdullah R. The influence of system quality characteristics on health care providers’ performance: empirical evidence from Malaysia. J Infect Public Health. 2016 Nov-Dec;9(6):698–707. DOI: 10.1016/j.jiph.2016.09.002. 27659115

[27] Zakaria N, Yusof SAM. Understanding technology and people issues in hospital information system (HIS) adoption: case study of a tertiary hospital in Malaysia. J Infect Public Health. 2016 Nov-Dec;9(6):774–80. DOI: 10.1016/j.jiph.2016.08.017. 27686258

[28] Househ MS, Kushniruk A, Maclure M, Carleton B, Cloutier-Fisher D. The use of conferencing technologies to support drug policy group knowledge exchange processes: an action case approach. Int J Med Inform. 2011 4;80(4):251–61. DOI: 10.1016/j.ijmedinf.2010.10.020. 21167772

[29] Mufti HN, Hirsch GM, Abidi SR, Abidi SSR. Exploiting machine learning algorithms and methods for the prediction of agitated delirium after cardiac surgery: models development and validation study. JMIR Med Inf. 2019 Oct-Dec;7(4):193–213. DOI: 10.2196/14993. PMC6913743 31558433

[30] Albarrak AI. Medical informatics in undergraduate medical study. Technol Health Care. 2005;13(5):356–7.

[31] Al-Hajjaj MS, Al-Khatim IM. High rate of non-compliance with anti-tuberculosis treatment despite a retrieval system: a call for implementation of directly observed therapy in Saudi Arabia. Int J Tuberc Lung Dis. 2000 4;4(4):345–9. 10777084

[32] Mitri W, Sandridge AL, Subhani S, Greer W. Design and development of an Internet registry for congenital heart defects. Teratology. 2002 2;65(2):78–87. DOI: 10.1002/tera.10016. 11857509

[33] Al-Safadi LAE, IEEE Computer Society. Semantic-based exchanger for electronic medical record. Los Alamitos, CA: The Society; 2008. 962–7 p. (Third 2008 International Conference on Convergence and Hybrid Information Technology, Vol. 1, proceedings). ISBN: 978-0-7695-3407-7.

[34] Abdel-Aal RE. Abductive network committees for improved classification of medical data. Methods Inf Med. 2004;43(2):192–201. 15136869

[35] Abdel-Aal RE. Improved classification of medical data using abductive network committees trained on different feature subsets. Comput Methods Programs Biomed. 2005 11;80(2):141–53. DOI: 10.1016/j.cmpb.2005.08.001. 16169631

[36] Alghamdi E, Yunus F, Househ M. Revisiting the impact of mobile phone screen size on user comprehension of health information. Stud Health Technol Inform. 2014;202:217–20. 25000055

[37] Househ M, Kushniruk A, Cloutier-Fisher D, Carleton B. Technology enabled knowledge exchange: development of a conceptual framework. J Med Syst. 2011 8;35(4):713–21. DOI: 10.1007/s10916-009-9408-0. 20703520

[38] Househ M, Alshammri R, Jradi H, Da’ar OB, Saddik B, Alamry A. Aligning public health and health informatics research strengths with national level research priorities in Saudi Arabia. Stud Health Technol Inform. 2014;202:249–52. 25000063

[39] Saddik B, Al-Mansour S. Does CPOE support nurse-physician communication in the medication order process? a nursing perspective. Stud Health Technol Inform. 2014;204:149–55. 25087542

[40] AlSaadi MM. Evaluation of Internet use for health information by parents of asthmatic children attending pediatric clinics in Riyadh, Saudi Arabia. Ann Saudi Med. 2012 Nov-Dec;32(6):630–6. DOI: 10.5144/0256-4947.2012.630. 23396028 PMC6081114

[41] Albarrak AI. Information security behavior among nurses in an academic hospital. HealthMED. 2012;6(7):2349–54.

[42] Saddik B, Al-Fridan MM. Physicians’ satisfaction with computerised physician order entry (CPOE) at the national guard health affairs: a preliminary study. Stud Health Technol Inform. 2012;178:199–206. 22797042

[43] Aldosari B. Rates, levels, and determinants of electronic health record system adoption: a study of hospitals in Riyadh, Saudi Arabia. Int J Med Inform. 2014 5;83(5):330–42. DOI: 10.1016/j.ijmedinf.2014.01.006. 24560609

[44] Walston SL, Mwachofi A, Aldosari B, Al-Omar BA, Yousef AA, Sheikh A. The impact of information technology and organizational focus on the visibility of patient care errors. Qual Manag Health Care. 2010 Jul-Sep;19(3):248–58. DOI: 10.1097/QMH.0b013e3181eb3b1d. 20588143

[45] Shaker HA, Farooq MU. Usability evaluation of paper-based “hajji” health record format. J Med Syst. 2012 4;36(2):853–63. DOI: 10.1007/s10916-010-9549-1. 20703648

[46] Bah S, Alharthi H, El Mahalli AA, Jabali A, Al-Qahtani M, Al-kahtani N. Annual survey on the level and extent of usage of electronic health records in government-related hospitals in eastern province, Saudi Arabia. Perspect Health Inf Manag. 2011 Fall;8:1b. PMC3193507 22016668

[47] BinDhim NF, McGeechan K, Trevena L. Assessing the effect of an interactive decision-aid smartphone smoking cessation application (app) on quit rates: a double-blind automated randomised control trial protocol. BMJ Open. 2014 7 18;4(7):e005371. DOI: 10.1136/bmjopen-2014-005371. PMC4120407 25037644

[48] Mwachofi A, Walston SL, Al-Omar BA. Factors affecting nurses’ perceptions of patient safety. Int J Health Care Qual Assur. 2011;24(4):274–83. DOI: 10.1108/09526861111125589. 21938973

[49] Almutairi MS, Alseghayyir RM, Al-Alshikh AA, Arafah HM, Househ MS. Implementation of computerized physician order entry (CPOE) with clinical decision support (CDS) features in Riyadh hospitals to improve quality of information. Stud Health Technol Inform. 2012;180:776–80. 22874297

[50] Alasmary M, El Metwally A, Househ M. The association between computer literacy and training on clinical productivity and user satisfaction in using the electronic medical record in Saudi Arabia education & training. J Med Syst. 2014 8;38(8):69. DOI: 10.1007/s10916-014-0069-2. 24957393

[51] AlGhamdi KM, Almohideb MA. Internet use by dermatology outpatients to search for health information. Int J Dermatol. 2011 3;50(3):292–9. DOI: 10.1111/j.1365-4632.2010.04705.x. 21342162

[52] AlGhamdi KM, Moussa NA. Internet use by the public to search for health-related information. Int J Med Inform. 2012 6;81(6):363–73. DOI: 10.1016/j.ijmedinf.2011.12.004. 22217800

[53] Alharthi H, Sultana N, Al-Amoudi A, Basudan A. An analytic hierarchy process-based method to rank the critical success factors of implementing a pharmacy barcode system. Perspect Health Inf Manag. 2015 1 1;12(Winter):1g. PMC4700872 26807079

[54] Atallah N, Khalifa M, El Metwally A, Househ M. The prevalence and usage of mobile health applications among mental health patients in Saudi Arabia. Comput Methods Programs Biomed. 2018 3;156:163–8. DOI: 10.1016/j.cmpb.2017.12.002. 29428068

[55] Khalifa M, Alswailem O. Clinical decision support knowledge management: strategies for success. Stud Health Technol Inform. 2015;213:67–70. 26152955

[56] Alshagathrh F, Khan SA, Alothmany N, Al-Rawashdeh N, Househ M. Building a cloud-based data sharing model for the Saudi national registry for implantable medical devices: results of a readiness assessment. Int J Med Inform. 2018 10;118:113–9. DOI: 10.1016/j.ijmedinf.2018.08.005. 30153916

[57] Aldosari B, Al-Mansour S, Aldosari H, Alanazi A. Assessment of factors influencing nurses acceptance of electronic medical record in a Saudi Arabia hospital. Inform Med Unlocked. 2018;10:82–8. DOI: 10.1016/j.imu.2017.12.007.

[58] El Mahalli A, El-Khafif SH, Yamani W. Assessment of pharmacy information system performance in three hospitals in eastern province, Saudi Arabia. Perspect Health Inf Manag. 2016 1 1;13(Winter):1b. PMC4739441 26903780

[59] Albarrak AI, Mohammed R, Almarshoud N, Almujalli L, Aljaeed R, Altuwaijiri S, Albohairy T. Assessment of physician's knowledge, perception and willingness of telemedicine in Riyadh region, Saudi Arabia. J Infect Public Health. 2021 1;14(1):97–102. DOI: 10.1016/j.jiph.2019.04.006. 31060975

[60] Al-Khathaami AM, Alshahrani SM, Kojan SM, Al-Jumah MA, Alamry AA, El-Metwally AA. Cultural acceptance of robotic telestroke medicine among patients and healthcare providers in Saudi Arabia: results of a pilot study. Neurosciences (Riyadh). 2015 1;20(1):27–30. 25630777 PMC4727601

[61] Aldosari B, Alanazi A. Documentation integrity: authorship functionalities of EHR in a Saudi Arabian hospital. Comput Biol Med. 2018 2 1;93:184–8. DOI: 10.1016/j.compbiomed.2017.12.015. 29324363

[62] Alshammari F. Factors influencing decisions to enroll in the health informatics educational programs. Inform Health Soc Care. 2016;41(2):177–91. DOI: 10.3109/17538157.2015.1008483. 25710093

[63] Alsohime F, Temsah MH, Al-Eyadhy A, Bashiri FA, Househ M, Jamal A, Hasan G, Alhaboob AA, Alabdulhafid M, Amer YS. Satisfaction and perceived usefulness with newly-implemented electronic health records system among pediatricians at a university hospital. Comput Methods Programs Biomed. 2019 2;169:51–7. DOI: 10.1016/j.cmpb.2018.12.026. 30638591

[64] Albarrak AI, Mohammed R, Zakaria N, Alyousef LM, Almefgai NB, Alqahtani HD, Alamer HS, Alsulaiman AA. The impact of obesity related websites on decision making among students in Saudi Arabia. Saudi Pharm J. 2016 9;24(5):605–10. DOI: 10.1016/j.jsps.2015.03.016. 27752234 PMC5059827

[65] Sayedalamin Z, Alshuaibi A, Almutairi O, Baghaffar M, Jameel T, Baig M. Utilization of smart phones related medical applications among medical students at King Abdulaziz University, Jeddah: a cross-sectional study. J Infect Public Health. 2016 Nov-Dec;9(6):691–7. DOI: 10.1016/j.jiph.2016.08.006. 27666637

[66] Al Muallem Y, Al Dogether M, Househ M, Saddik B. Auditing the completeness and legibility of computerized radiological request forms. J Med Syst. 2017 11 4;41(12):199. DOI: 10.1007/s10916-017-0826-0. 29101478

[67] Alahmad G, Hifnawy T, Abbasi B, Dierickx K. Attitudes toward medical and genetic confidentiality in the Saudi research biobank: an exploratory survey. Int J Med Inform. 2016 3 87:84–90. DOI: 10.1016/j.ijmedinf.2015.12.015. 26806715

[68] Shaikh A. The impact of SOA on a system design for a telemedicine healthcare system. Netw Model Analy Health Inform Bioinform. 2015;4(15). DOI: 10.1007/s13721-015-0087-0.

[69] Justinia T, Alyami A, Al-Qahtani S, Bashanfar M, El-Khatib M, Yahya A, Zagzoog F. Social media and the orthopaedic surgeon: a mixed methods study. Acta Inform Med. 2019 3;27(1):23–8. DOI: 10.5455/aim.2019.27.23-28. 31213739 PMC6511278

[70] Alalawi ZM, Eid MM, Albarrak AI. Assessment of picture archiving and communication system (PACS) at three of ministry of health hospitals in Riyadh region – content analysis. J Infect Public Health. 2016 Nov-Dec;9(6):713–24. DOI: 10.1016/j.jiph.2016.09.004. 27659113

[71] Almaiman S, Bahkali S, Al Farhan A, Bamuhair S, Househ M, Alsurimi K. The prevalence of using social media among healthcare professionals in Saudi Arabia: a pilot study. Stud Health Technol Inform. 2015;213:263–6. 26153010

[72] Alqahtani AS, BinDhim NF, Tashani M, Willaby HW, Wiley KE, Heywood AE, Booy R, Rashid H. Pilot use of a novel smartphone application to track traveller health behaviour and collect infectious disease data during a mass gathering: hajj pilgrimage 2014. J Epidemiol Glob Health. 2016 9;6(3):147–55. DOI: 10.1016/j.jegh.2015.07.005. 26279527 PMC7104231

[73] BinDhim NF, Shaman AM, Trevena L, Basyouni MH, Pont LG, Alhawassi TM. Depression screening via a smartphone app: cross-country user characteristics and feasibility. J Am Med Inform Assoc. 2015 1;22(1):29–34. DOI: 10.1136/amiajnl-2014-002840. 25326599 PMC4433364

[74] Ayaad O, Alloubani A, ALhajja EA, Farhan M, Abuseif S, Al Hroub A, Akhu-Zaheya L. The role of electronic medical records in improving the quality of health care services: comparative study. Int J Med Inform. 2019 7;127:63–7. DOI: 10.1016/j.ijmedinf.2019.04.014. 31128833

[75] Al Ghamdi E, Yunus F, Da’ar O, El-Metwally A, Khalifa M, Aldossari B, Househ M. The effect of screen size on mobile phone user comprehension of health information and application structure: an experimental approach. J Med Syst. 2016 1;40(1):1–8. DOI: 10.1007/s10916-015-0381-5. 26573648

[76] Onezi HA, Khalifa M, El-Metwally A, Househ M. The impact of social media-based support groups on smoking relapse prevention in Saudi Arabia. Comput Methods Programs Biomed. 2018 6;159:135–43. DOI: 10.1016/j.cmpb.2018.03.005. 29650308

[77] Jamal A, Khan SA, AlHumud A, Al-Duhyyim A, Alrashed M, Bin Shabr F, Alteraif A, Almuziri A, Househ M, Qureshi R. Association of online health information-seeking behavior and self-care activities among type 2 diabetic patients in Saudi Arabia. J Med Internet Res. 2015 8;17(8):15. DOI: 10.2196/jmir.4312. PMC4642387 26268425

[78] Brieux HFM, Benitez S, Otero C, Luna D, Masud JHB, Marcelo A, Househ M, Hullin C, Villalba C, Indarte S, Guillen S, Otero P, Campos F, Baum A, de Quiros FGB. Cultural problems associated with the implementation of ehealth. In: Gundlapalli AV, Jaulent MC, Zhao D, eds. Medinfo 2017: precision healthcare through informatics: studies in health technology and informatics. Vol. 245. Amsterdam, Netherlands: IOS Press; 2017. p. 1213. 29295300

[79] Almutairi A, Mc Crindle PR, IEEE. A pilot study in Jeddah city of nurses perceptions of electronic medical records. New York, NY: IEEE; 2015. p. 1054–6. (2015 SAI Intelligent Systems Conference). ISBN: 978-1-4673-7606-8.

[80] Zaman TU, Raheem TMA, Alharbi GM, Shodri MF, Kutbi AH, Alotaibi SM, Aldaadi KS. E-health and its transformation of healthcare delivery system in Makkah, Saudi Arabia. Int J Med Res Health Sci. 2018;7(5):76–82.

[81] Khalifa M. Evaluating nurses acceptance of hospital information systems: a case study of a tertiary care hospital. In: Sermeus W, Procter PM, Weber P, eds. Nursing informatics 2016: ehealth for all: every level collaboration - from project to realization: studies in health technology and informatics. Vol. 225. Amsterdam, Netherlands: IOS Press; 2016. p. 78–82. 27332166

[82] Alharbi AH. A portable virtual lab for informatics education using open source software. Int J Adv Comput Sci Appl. 2018 2;9(2):142–7.

[83] Bahkali S, Almaiman R, El-Awad M, Almohanna H, Al-Surimi K, Househ M. Exploring the impact of information seeking behaviors of online health consumers in the Arab world. In: Mantas J, Hasman A, Gallos P, Kolokathi A, Househ MS, eds. Unifying the applications and foundations of biomedical and health informatics: studies in health technology and informatics. Vol. 226. Amsterdam, Netherlands: IOS Press; 2016. p. 279–82. 27350525

[84] Almutairi A, McCrindle R,, IEEE. Female student nurses attitudes towards electronic medical records in Riyadh city. New York, NY: IEEE; 2016. p. 2366–70. (2016 International Conference on Electrical, Electronics, and Optimization Techniques). ISBN: 978-1-4673-9939-5.

[85] Meri A, Hasan MK, Danaee M, Jaber M, Jarrar M, Safei N, Dauwed M, Abd SK, Al-Bsheish M. Modelling the utilization of cloud health information systems in the Iraqi public healthcare sector. Telemat Inform. 2019 3;36:132–46. DOI: 10.1016/j.tele.2018.12.001.

[86] Bahkali S, Alkharjy N, Owairdy MA, Househ M, Da’ar O, Alsurimi K. A social media campaign to promote breastfeeding among Saudi women: a web-based survey study. In: Mantas J, Hasman A, Gallos P, Kolokathi A, Househ MS, eds. Enabling health informatics applications: studies in health technology and informatics. Vol. 213. Amsterdam, Netherlands: IOS Press; 2015. p. 247–50. 26153006

[87] Ahmed A. Nursing informatics competencies among nursing students and their relationship to patient safety competencies knowledge, attitude, and skills. Comput Inform Nurs. 2015 11;33(11):509–14. DOI: 10.1097/cin.0000000000000197. 26524185

[88] Almaiman S, Bahkali S, Alabdulatif N, Bahkaly A, Al-Surimi K, Househ M. Promoting oral health using social media platforms: seeking Arabic online oral health related information (OHRI). In: Mantas J, Hasman A, Gallos P, Kolokathi A, Househ MS, eds. Unifying the applications and foundations of biomedical and health informatics: studies in health technology and informatics. Vol. 226. Amsterdam, Netherlands: IOS Press; 2016. p. 283–6. 27350526

[89] Khalifa M, Zabani I. Reducing emergency department crowding: evidence based strategies. In: Mantas J, Hasman A, Gallos P, Kolokathi A, Househ MS, eds. Unifying the applications and foundations of biomedical and health informatics: studies in health technology and informatics. Vol. 226. Amsterdam, Netherlands: IOS Press; 2016. p. 67–70. 27350468

[90] Alenazi H, Alghamdi M, Alradhi S, Househ M, Zakaria N. A study on Saudi diabetic patients’ readiness to use mobile health. In: Gundlapalli AV, Jaulent MC, Zhao D, eds. Medinfo 2017: precision healthcare through informatics: studies in health technology and informatics. Vol. 245. Amsterdam, Netherlands: IOS Press; 2017. p. 1210. 29295297

[91] AlOthman R, Zakaria N, AlBarrak A. Saudi diabetic patients’ attitudes towards patient portal use and their perceived e-health literacy. In: Gundlapalli AV, Jaulent MC, Zhao D, eds. Medinfo 2017: precision healthcare through informatics: studies in health technology and informatics. Vol. 245. Amsterdam, Netherlands: IOS Press; 2017. p. 1211. 29295298

[92] Bahkali S, Alfurih S, Aldremly M, Alzayyat M, Alsurimi K, Househ M. The prevalence of Internet and social media based medication information seeking behavior in Saudi Arabia. In: Mantas J, Hasman A, Gallos P, Kolokathi A, Househ MS, eds. Unifying the applications and foundations of biomedical and health informatics: studies in health technology and informatics. Vol. 226. Amsterdam, Netherlands: IOS Press; 2016. p. 275–8. 27350524

[93] Aldosari B. User acceptance of a picture archiving and communication system (PACS) in a Saudi Arabian hospital radiology department. BMC Med Inform Decis Mak. 2012 5;12:10. DOI: 10.1186/1472-6947-12-44. 22640490 PMC3423046

[94] Khalifa M, Alswailem O. Clinical pathways: identifying development, implementation and evaluation challenges. In: Mantas J, Hasman A, Househ MS, eds. Enabling health informatics applications: studies in health technology and informatics. Vol. 213. Amsterdam, Netherlands: IOS Press; 2015. p. 131–4. 26152973

[95] Alaboudi A, Atkins A, Sharp B, Balkhair A, Alzahrani M, Sunbul T. Barriers and challenges in adopting Saudi telemedicine network: the perceptions of decision makers of healthcare facilities in Saudi Arabia. J Infect Public Health. 2016 Nov-Dec;9(6):725–33. DOI: 10.1016/j.jiph.2016.09.001. 27649882

[96] Tesfaye B, Atique S, Azim T, Kebede MM. Predicting skilled delivery service use in Ethiopia: dual application of logistic regression and machine learning algorithms. BMC Med Inform Decis Mak. 2019 11;19(1):10. DOI: 10.1186/s12911-019-0942-5. 31690306 PMC6833149

[97] Al Saleem N, El Metwally A, Househ M. Electronic lab information exchange (ELIE) in Saudi Arabia. Stud Health Technol Inform. 2014;202:134–7. 25000034

[98] Bahkali S, Almaiman A, Almadani W, Househ M, El Metwally A. The state public health informatics in Saudi Arabia. Stud Health Technol Inform. 2014;202:257–60. 25000065

[99] Almaiman A, Bahkali S, Alfrih S, Househ M, El Metwally A. The use of health information technology in Saudi primary healthcare centers. Stud Health Technol Inform. 2014;202:209–12. 25000053

[100] Al-Nasser L, Al-Ehaideb A, Househ M. Assessing the current state of dental informatics in Saudi Arabia: the new frontier. Stud Health Technol Inform. 2014;202:165–8. 25000042

[101] Almaiman A, Bahkali S, Bahkali A, Almaiman S, Elmetwally A, Househ M. Electronic dental record (EDR) use in Saudi Arabia: an exploratory study. Stud Health Technol Inform. 2014;202:169–72. 25000043

[102] Al Saleem N, Househ M, El Metwally A. Challenges in building health surveillance systems in Saudi Arabia. Stud Health Technol Inform. 2014;202:261–4. 25000066

[103] Alanazi A, Anazi YA. The challenges in personal health record adoption. J Healthc Manag. 2019 Mar-Apr;64(2):104–9. DOI: 10.1097/JHM-D-17-00191. 30845058

[104] Aldosari B. Causes of EHR projects stalling or failing: a study of EHR projects in Saudi Arabia. Comput Biol Med. 2017 12;91:372–81. DOI: 10.1016/j.compbiomed.2017.10.032. 29127903

[105] Al Ateeq A, Al Moamary E, Daghestani T, Al Muallem Y, Al Dogether M, Alsughayr A, Altuwaijri M, Househ M. Using a digital marketing platform for the promotion of an Internet based health encyclopedia in Saudi Arabia. Stud Health Technol Inform. 2015;208:12–6. 25676939

[106] Aldosari B. Supportive care pathway functionalities of EHR system in a Saudi Arabian hospital. Comput Biol Med. 2017 10; 89:190–6. DOI: 10.1016/j.compbiomed.2017.08.012. 28822900

[107] Al Muallem Y, Al Dogether M, Al Assaf R, Al Ateeq A, Househ M. A pharmacy inventory management system in Saudi Arabia: a case study. Stud Health Technol Inform. 2015;208:17–21. 25676940

[108] Al Dogether M, Al Muallem Y, Al Assaf R, Al Ateeq A, Al Moammary E, Al Ghamdi H, Househ M. The implementation experiences of an endoscopy information system (EIS) on the improvement of workflow processes in a Saudi endoscopy department. Stud Health Technol Inform. 2015;213:29–32. 26152945

[109] Al Muallem Y, Al Dogether M, Al Assaf R, Al Ateeq A, Househ M. The implementation experiences of a pharmacy automation drug dispensing system in Saudi Arabia. Stud Health Technol Inform. 2015;208:22–6. 25676941

[110] Alsulame K, Khalifa M, Househ M. Ehealth in Saudi Arabia: current trends, challenges and recommendations. Stud Health Technol Inform. 2015;213:233–6. 26153002

[111] Alsharif S, Benslimane N, Khalifa M, Price C. Healthcare IT strategic alignment: challenges and recommendations. Stud Health Technol Inform. 2018;251:207–10. 29968639

[112] Aldosari B. Health ATMs in Saudi Arabia: a perspective. Acta Inform Med. 2017 6;25(2):130–5. DOI: 10.5455/aim.2017.25.130-135. 28883680 PMC5544459

[113] Househ MS, Al-Tuwaijri M. Early development of an enterprise health data warehouse. In: Borycki EM, BartleClar JA, Househ MS, Kuziemsky CE, Schraa EG, eds. International perspectives in health informatics: studies in health technology and informatics. Vol. 164. Amsterdam, Netherlands: IOS Press; 2011. p. 122–6. 21335698

[114] Bahkali S, Almaiman A, Altassan N, Almaiman S, Househ M, Alsurimi K. Exploring the role of Twitter in promoting women's health in the Arab world: lessons learned. In: Mantas J, Hasman A, Househ MS, eds. Enabling health informatics applications: studies in health technology and informatics. Vol. 213. Amsterdam, Netherlands: IOS Press; 2015. p. 251–4. 26153007

[115] Barakah DM. Integrating dental working experience in development of a dental clinic database system for a general tertiary hospital. In: Arabnia HR, Deligiannidis L, Yang M, eds. New York, NY: IEEE; 2016. p. 63–7. (2016 International Conference on Computational Science & Computational Intelligence). ISBN: 978-1-5090-5510-4.

[116] Kedwan FH, Justinia T. Patients online registration system: feasibility and perceptions. Ann Med Health Sci Res. 2017 11;7:90–5.

[117] Al Muallem Y, Al Dogether M, Al Assaf R, Al Ateeq A, Al Moammary E, Al Ghamdi H, Househ M. Remote patient monitoring system implementation at a cardiac care centre in Saudi Arabia. In: Mantas J, Hasman A, Househ MS, eds. Enabling health informatics applications: studies in health technology and informatics. Vol. 213. Amsterdam, Netherlands: IOS Press; 2015. p. 33–6. 26152946

[118] Al Muallem Y, Al Dogether M, Al Ateeq A, Al Moammary E, Al Ghamdi H, Almeshari M, Househ M. Vagal nerve stimulation (VNS) therapy system implementation at a neurology department in Saudi Arabia. In: Mantas J, Hasman A, Househ MS, eds. Enabling health informatics applications: studies in health technology and informatics. Vol. 213. Amsterdam, Netherlands: IOS Press; 2015. p. 37–40. 26152947

[119] Binobaid S, Fan IS, Almeziny M. Investigation interoperability problems in pharmacy automation: a case study in Saudi Arabia. In: Varajao JEQ, CruzCunha MM, Martinho R, Rijo R, Bjorn-Andersen N, Turner R, Alves D, eds. International Conference on Enterprise Information Systems/International Conference on Project Management/International Conference on Health and Social Care Information Systems and Technologies, Centeris/Projman/HCIST 2016. Procedia Computer Science. Vol. 100. Amsterdam: Elsevier Science; 2016. p. 329–38.

[120] Altuwaijri MM, Sughayr AM, Hassan MA, AlAzwari FM. The effect of integrating short messaging services reminders with electronic medical records on non-attendance rates. Saudi Med J. 2012 2;33(2):193–6. 22327762

[121] Al-Dorzi HM, Tamim HM, Cherfan A, Hassan MA, Taher S, Arabi YM. Impact of computerized physician order entry (CPOE) system on the outcome of critically ill adult patients: a before-after study. BMC Med Inform Decis Mak. 2011 11 19;11:71. DOI: 10.1186/1472-6947-11-71. 22098683 PMC3248372

[122] Mominah MA, Househ MS. Identifying computerized provider order entry (CPOE) medication errors. Stud Health Technol Inform. 2013;190:210–2. 23823425

[123] Subhani S, Al-Rubeaan K. Design and development of a web-based Saudi national diabetes registry. J Diabetes Sci Technol. 2010 11 1;4(6):1574–82. DOI: 10.1177/193229681000400635. 21129356 PMC3005071

[124] Daghistani TA, Elshawi R, Sakr S, Ahmed AM, Al-Thwayee A, Al-Mallah MH. Predictors of in-hospital length of stay among cardiac patients: a machine learning approach. Int J Cardiol. 2019 8 1;288:140–7. DOI: 10.1016/j.ijcard.2019.01.046. 30685103

[125] Daghistani T, Shammari RA, Razzak MI. Discovering diabetes complications: an ontology based model. Acta Inform Med. 2015 12;23(6):385–92. DOI: 10.5455/aim.2015.23.385-392. 26862251 PMC4720828

[126] Househ MS, Shubair MM, Yunus F, Jamal A, Aldossari B. The use of an adapted health it usability evaluation model (health-ituem) for evaluating consumer reported ratings of diabetes mhealth applications: implications for diabetes care and management. Acta Inform Med. 2015 10;23(5):290–5. DOI: 10.5455/aim.2015.23.290-295. 26635437 PMC4639336

[127] Al-Jiffry BO, Khayat S, Abdeen E, Hussain T, Yassin M. A scoring system for the prediction of choledocholithiasis: a prospective cohort study. Ann Saudi Med. 2016 Jan-Feb;36(1):57–63. DOI: 10.5144/0256-4947.2016.57. 26922689 PMC6074271

[128] Djemal R, AlShArabi K, Ibrahim S, Alsuwailem A. EEG-based computer aided diagnosis of autism spectrum disorder using wavelet, entropy, and ANN. Biomed Res Int. 2017;2017:9816591. DOI: 10.1155/2017/9816591. 28484720 PMC5412163

[129] Shuaib QM, Vijey T. Self-optimized routing algorithm for mhealth and remote health monitoring. J Med Imaging Health Inform. 2016 2;6(1):189–93. DOI: 10.1166/jmihi.2016.1597.

[130] AlMuhaideb S, Alswailem O, Alsubaie N, Ferwana I, Alnajem A. Prediction of hospital no-show appointments through artificial intelligence algorithms. Ann Saudi Med. 2019 Nov-Dec;39(6):373–81. DOI: 10.5144/0256-4947.2019.373. 31804138 PMC6894458

[131] Zheng G, Fang G, Shankaran R, Orgun MA, Zhou J, Qiao L, Saleem K. Multiple ECG fiducial points-based random binary sequence generation for securing wireless body area networks. IEEE J Biomed Health Inform. 2017 5;21(3):655–63. DOI: 10.1109/JBHI.2016.2546300. 27046882

[132] Khalifa M, Zabani I. Utilizing health analytics in improving the performance of healthcare services: a case study on a tertiary care hospital. J Infect Public Health. 2016 Nov-Dec;9(6):757–65. DOI: 10.1016/j.jiph.2016.08.016. 27663517

[133] Seyam RM, Alalawi MM, Alkhudair WK, Alzahrani HM, Azhar RA, Alothman KI, Al-Hussain TO, Alotaibi MF. Operative outcomes of robotic partial nephrectomy. a report of the first 101 cases from a single center in Saudi Arabia. Saudi Med J. 2019 1;40(1):33–40. DOI: 10.15537/smj.2019.1.22782. 30617378 PMC6452606

[134] Ali Z, Alsulaiman M, Muhammad G, Al-nasheri A, Mahmood A,, IEEE. Clinical informatics: mining of pathological data by acoustic analysis. New York, NY: IEEE; 2017. (2017 International Conference on Informatics, Health & Technology). ISBN: 978-1-4673-8765-1.

[135] Sakr S, Elshawi R, Ahmed AM, Qureshi WT, Brawner CA, Keteyian SJ, Blaha MJ, Al-Mallah MH. Comparison of machine learning techniques to predict all-cause mortality using fitness data: the Henry Ford exercise testing (fit) project. BMC Med Inform Decis Mak. 2017 12;17:15. DOI: 10.1186/s12911-017-0566-6. 29258510 PMC5735871

[136] Khalifa M. Developing an emergency physician productivity index using descriptive health analytics. In: Mantas J, Hasman A, Househ MS, eds. Enabling health informatics applications: studies in health technology and informatics. Vol. 213. Amsterdam, Netherlands: IOS Press; 2015. p. 167–70. 26152983

[137] Alsolamy S, Al Salamah M, Al Thagafi M, Al-Dorzi HM, Marini AM, Aljerian N, Al-Enezi F, Al-Hunaidi F, Mahmoud AM, Alamry A, Arabi YM. Diagnostic accuracy of a screening electronic alert tool for severe sepsis and septic shock in the emergency department. BMC Med Inform Decis Mak. 2014 12;14:105. DOI: 10.1186/s12911-014-0105-7. 25476738 PMC4261595

[138] Khalifa M, Zabani I, Khalid P. Exploring lab tests over utilization patterns using health analytics methods. In: Mantas J, Hasman A, Gallos P, Kolokathi A, Househ MS, eds. Unifying the applications and foundations of biomedical and health informatics: studies in health technology and informatics. 226. Amsterdam, Netherlands: IOS Press; 2016. p. 190–3. 27350501

[139] Alsalamah S, Gray WA, Hilton J, Alsalamah H. Information security requirements in patient-centred healthcare support systems. In: Lehmann CU, Ammenwerth E, Nohr C, eds. Medinfo 2013: Proceedings of the 14th World Congress on Medical and Health Informatics, parts 1 and 2: studies in health technology and informatics. Vol. 192. Amsterdam, Netherlands: IOS Press; 2013. p. 812–6. 23920670

[140] Gonsalves AH, Thabtah F, Mohammad RMA, Singh G; Association for Computing Machinery. Prediction of coronary heart disease using machine learning: an experimental analysis. New York, NY: Association for Computing Machinery; 2019. p. 51–6 (ICDLT 2019: 2019 3rd International Conference on Deep Learning Technologies). ISBN: 978-1-4503-7160-5.

[141] Khalifa M, Khalid P. Reducing unnecessary laboratory testing using health informatics applications: a case study on a tertiary care hospital. In: Shakshuki EM, ed. 5th International Conference on Emerging Ubiquitous Systems and Pervasive Networks/4th International Conference on Current and Future Trends of Information and Communication Technologies in Healthcare/Affiliated Workshops. Procedia Computer Science. 37. Amsterdam, Netherlands: Elsevier Science; 2014. p. 253–60.

[142] Khalifa M. Utilizing health analytics in improving emergency room performance. In: Sermeu W, Procter PM, Weber P, eds. Nursing informatics 2016: ehealth for all: every level collaboration - from project to realization: studies in health technology and informatics. Vol. 225. Amsterdam, Netherlands: IOS Press; 2016. p. 138–42. 27332178

[143] Almazyad AS, Ahamad MG, Siddiqui MK, Almazyad AS. Effective hypertensive treatment using data mining in Saudi Arabia. J Clin Monit Comput. 2010 12;24(6):391–401. DOI: 10.1007/s10877-010-9260-2. 20978930

[144] Kattan WM, Abduljawad AA. Predicting different factors that affect hospital utilization and outcomes among diabetic patients admitted with hypoglycemia using structural equation modeling. Diabetes Res Clin Pract. 2019 7;153:55–65. DOI: 10.1016/j.diabres.2019.05.031. 31152808

[145] Alsomali W, Razzak I, Alshammari R. Development of ontology for penicillin-related adverse events. J Med Imaging Health Inform. 2016 6;6(3):620–6. DOI: 10.1166/jmihi.2016.1724.

[146] Noor A, Assiri A, Ayvaz S, Clark C, Dumontier M. Drug-drug interaction discovery and demystification using semantic web technologies. J Am Med Inform Assoc. 2017 5;24(3):556–64. DOI: 10.1093/jamia/ocw128. 28031284 PMC7651897

[147] Nasir M, Anjum A, Manzoor U, Balubaid MA, Ahmed M, Khan A, Ahmad N, Malik SUR, Alam M. Privacy preservation in skewed data using frequency distribution and weightage (FDW). J Med Imaging Health Inform. 2017 10;7(6):1346–57. DOI: 10.1166/jmihi.2017.2206.

[148] Moqurrab SA, Anjum A, Manzoor U, Nefti S, Ahmad N, Malik SUR. Differential average diversity: an efficient privacy mechanism for electronic health records. J Med Imaging Health Inform. 2017 10;7(6):1177–87. DOI: 10.1166/jmihi.2017.2146.

[149] Mughal B, Sharif M, Muhammad N, Saba T. A novel classification scheme to decline the mortality rate among women due to breast tumor. Microsc Res Tech. 2018 2;81(2):171–80. DOI: 10.1002/jemt.22961. 29143395

[150] Orozco-Arroyave JR, Belalcazar-Bolanos EA, Arias-Londono JD, Vargas-Bonilla JF, Skodda S, Rusz J, Daqrouq K, Honig F, Noth E. Characterization methods for the detection of multiple voice disorders: neurological, functional, and laryngeal diseases. IEEE J Biomed Health Inform. 2015 11;19(6):1820–8. DOI: 10.1109/jbhi.2015.2467375. 26277012

[151] Iliyasu AM, Fatichah C, Abuhasel KA. Evidence accumulation clustering with possibilitic fuzzy c-means base clustering approach to disease diagnosis. Automatika. 2016;57(3):822–35. DOI: 10.7305/automatika.2016.10.1427.

[152] Medrano C, Igual R, Plaza I, Castro M, Fardoun HM, IEEE. Personalizable smartphone application for detecting falls. New York, NY: IEEE; 2014. p. 169–72 (2014 IEEE-EMBS International Conference on Biomedical and Health Informatics). ISBN: 978-1-4799-2131-7.

[153] Alharbi A, Bulpitt A, Johnson OA. Towards unsupervised detection of process models in healthcare. In: Ugon A, Karlsson D, Klein GO, Moen A, eds. Building continents of knowledge in oceans of data: the future of co-created ehealth: studies in health technology and informatics. Vol. 247. Amsterdam, Netherlands: IOS Press; 2018. p. 381–5. 29677987

[154] Thalib L, Furuya-Kanamori L, AlHabib KF, Alfaleh HF, AlShamiri MQ, Amin H, Al Suwaidi J, Sulaiman K, Almahmeed W, Alsheikh-Ali AA, Al-Motarreb A, Doi SA. Validation of the 6-month grace score in predicting 1-year mortality of patients with acute coronary syndrome admitted to the Arabian Gulf hospitals. Angiology. 2017 3;68(3):251–6. DOI: 10.1177/0003319716659179. 27432444

[155] Al-Surimi K, Househ M, Almohandis E, Alshagathrh F. Establishing a national medical device registry in Saudi Arabia: lessons learned and future work. In: Mantas J, Hasman A, Househ MS, eds. Enabling health informatics applications: studies in health technology and informatics. Vol. 213. Amsterdam, Netherlands: IOS Press; 2015. p. 23–6. 26152943

[156] Asiri E, Khalifa M, Shabir SA, Hossain MN, Iqbal U, Househ M. Sharing sensitive health information through social media in the Arab world. Int J Qual Health Care. 2017 2;29(1):68–74. DOI: 10.1093/intqhc/mzw137. 28003369

[157] AlQarni ZA, Yunus F, Househ MS. Health information sharing on Facebook: an exploratory study on diabetes mellitus. J Infect Public Health. 2016 Nov-Dec;9(6):708–12. DOI: 10.1016/j.jiph.2016.08.015. 27618634

[158] Da’ar OB, Yunus F, Md. Hossain N, Househ M. Impact of Twitter intensity, time, and location on message lapse of bluebird's pursuit of fleas in Madagascar. J Infect Public Health. 2017 Jul-Aug;10(4):396–402. DOI: 10.1016/j.jiph.2016.06.011. 27423931

[159] Bahkali S, Almaiman A, Bahkali A, Almaiman S, Househ M, Alsurimi K. The role of social media in promoting women's health education in Saudi Arabia. Stud Health Technol Inform. 2015;213:259–62. 26153009

[160] Carmona S, Alayed N, Al-Ibrahim A, D’Souza R. Realizing the potential of real-time clinical collaboration in maternal–fetal and obstetric medicine through Whatsapp. Obstet Med. 2018 6;11(2):83–9. DOI: 10.1177/1753495X18754457. 29997691 PMC6038020

[161] Hossain N, Househ M. Using Healthmap to analyse Middle East respiratory syndrome (MERS) data. In: Mantas J, Hasman A, Gallos P, Kolokathi A, Househ MS, eds. Unifying the applications and foundations of biomedical and health informatics: studies in health technology and informatics. Vol. 226. Amsterdam, Netherlands: IOS Press; 2016. p. 213–6. 27350507

[162] Sittig DF, Sengupta S, al-Daig H, Payne TH, Pincetl P. The role of the information architect at King Faisal specialist hospital and research centre. Proc Annu Symp Comput Appl Med Care. 1995:756–60. 8563391 PMC2579195

[163] Dutta S, Lanvin B, Paua F. The global information technology report 2003–2004. World Bank; 2003.

[164] Yergens DW, Tam-Tham H, Minty EP. Visualization of the IMIA yearbook of medical informatics publications over the last 25 years. Yearb Med Inform. 2016 6 30;suppl 1:S130–8. DOI: 10.15265/IYS-2016-s003. 27362591 PMC5171502

[165] Mesallam TA, Farahat M, Malki KH, Alsulaiman M, Ali Z, Al-Nasheri A, Muhammad G. Development of the Arabic voice pathology database and its evaluation by using speech features and machine learning algorithms. J Healthc Eng. 2017 2017:8783751. DOI: 10.1155/2017/8783751. 29201333 PMC5672151

[166] Thabtah F, Kamalov F, Rajab K. A new computational intelligence approach to detect autistic features for autism screening. Int J Med Inform. 2018 9;117:112–24. DOI: 10.1016/j.ijmedinf.2018.06.009. 30032959

[167] Al-Turaiki I, Alshahrani M, Almutairi T. Building predictive models for MERS-COV infections using data mining techniques. J Infect Public Health. 2016 Nov-Dec;9(6):744–8. DOI: 10.1016/j.jiph.2016.09.007. 27641481 PMC7102847

[168] Alharthi H. Healthcare predictive analytics: an overview with a focus on Saudi Arabia. J Infect Public Health. 2018 Nov-Dec;11(6):749–56. DOI: 10.1016/j.jiph.2018.02.005. 29526444

[169] Al-Surimi K, Khalifa M, Bahkali S, El-Metwally A, Househ M. The potential of social media and Internet-based data in preventing and fighting infectious diseases: from Internet to Twitter. Emerging and Re-Emerging Viral Infections. Adv Exp Med Biol. 2017;972:131–9. 28004307 10.1007/5584_2016_132 PMC7120659

[170] Alharbi I, Alyoubi B, Hoque MR, Almazmomi N. Big data based m-health application to prevent health hazards: a design science framework. Telemed J E Health. 2019 4;25(4):326–31. DOI: 10.1089/tmj.2018.0063. 30192202

[171] Justinia T. Blockchain technologies: opportunities for solving real-world problems in healthcare and biomedical sciences. Acta Inform Med. 2019 12;27(4):284–91. DOI: 10.5455/aim.2019.27.284-291. 32055097 PMC7004292

[172] Elhussein M, Gull H, Alobaid A, Ajez A, Aldulaijan R, Alasfoor M, Algaraawi N, IEEE. Big data framework for health informatics a solution for influenza surveillance in Saudi Arabia. New York, NY: IEEE; 2018. (2018 21st Saudi Computer Society National Computer Conference). ISBN: 978-1-5386-4110-1.

[173] Stier S, Breuer J, Siegers P, Thorson K. Integrating survey data and digital trace data: key issues in developing an emerging field. Soc Sci Comput Rev. 2020;38(5):503–16. DOI: 10.1177/0894439319843669.

[174] Fang R, Pouyanfar S, Yang Y, Chen S-C, Iyengar SS. Computational health informatics in the big data age: a survey. ACM Comput Surv. 2016;49(1):Article 12. DOI: 10.1145/2932707.

[175] Saudi Data & Artificial Intelligence Authority (SDAIA) [Internet]. The Authority [cited 29 Jan 2021]. <https://sdaia.gov.sa/>. 10.2991/jegh.k.210405.001 PMC8242113 33876596

[176] Altuwaijri MM. Electronic-health in Saudi Arabia. just around the corner? Saudi Med J. 2008 2;29(2):171–8. 18246222

[177] Al-Bishri J. Evaluation of biomedical research in Saudi Arabia. Saudi Med J. 2013 9;34(9):954–9. 24043009

[178] Jahan S, Al-Saigul AM. Primary health care research in Saudi Arabia: a quantitative analysis. Int J Health Sci. 2017 Apr-Jun;11(2):9–15. PMC5426411 28539857

[179] Nadri H, Rahimi B, Timpka T, Sedghi S. The top 100 articles in the medical informatics: a bibliometric analysis. J Med Syst. 2017 8 19;41(10):150. DOI: 10.1007/s10916-017-0794-4. 28825158

