# Supplementary material for: Health informatics publication trends in Saudi Arabia: a bibliometric analysis over the last twenty-four years
Source: J Med Libr Assoc. 2021 Apr 1;109(2):219–39. doi: 10.5195/jmla.2021.1072 (PMC8270356; doi:10.5195/jmla.2021.1072)
Supplement: Supplementary file 1 — Appendix A: Keywords search queries [file jmla-109-2-219-s01.docx]

# Health informatics publication trends in Saudi Arabia: a bibliometric analysis over the last twenty-four years

## Samar Binkheder; Raniah Aldekhyyel; Jwaher Almulhem

### APPENDIX A

### Keywords search queries

We searched for health informatics (HI) terms using Medical Subject Headings (MeSH) in PubMed and Emtree in Embase. If MeSH or Emtree keywords were not available, HI keywords (health informatics, population informatics, global health informatics) were used as text keywords in search queries in the three databases.

| Informatics discipline | Database | Searching terms and keywords | Number of retrieved publications |
| --- | --- | --- | --- |
| Health informatics | PubMed | (health informatics[Text Word] ) AND (Saudi[Text Word] OR Saudi Arabia[MeSH Terms]) | 19 |
|  | Embase | ('health informatics') AND ('Saudi Arabia'/exp OR 'Saudi') | 409 |
|  | Web of Science | "health informatics" AND (Saudi OR "Saudi Arabia") | 213 |
| Medical informatics | PubMed | (medical informatics[MeSH Terms] OR medical informatics[Text Word]) AND (Saudi[Text Word] OR Saudi Arabia[MeSH Terms]) | 223 |
|  | Embase | ('medical informatics'/exp OR 'medical informatics') AND ('saudi arabia'/exp OR ‘Saudi’) | 213 |
|  | Web of Science | "medical informatics" AND (Saudi OR "Saudi Arabia") | 50 |
| Nursing informatics | PubMed | (nursing informatics[MeSH Terms] OR nursing informatics[Text Word] ) AND (Saudi[Text Word] OR Saudi Arabia[MeSH Terms]) | 1 |
|  | Embase | ( 'nursing informatics'/exp OR 'nursing informatics') AND ('Saudi Arabia'/exp OR 'Saudi') | 1 |
|  | Web of Science | "nursing informatics" AND (Saudi OR "Saudi Arabia") | 5 |
| Dental informatics | PubMed | (dental informatics[MeSH Terms] OR dental informatics[Text Word]) AND (Saudi[Text Word] OR Saudi Arabia[MeSH Terms]) | 2 |
|  | Embase | ('dental informatics'/exp OR 'dental informatics') AND ('Saudi Arabia'/exp OR 'Saudi') | 0 |
|  | Web of Science | "dental informatics" AND (Saudi OR "Saudi Arabia") | 3 |
| Consumer health informatics | PubMed | (consumer health informatics[MeSH Terms] OR consumer health informatics[Text Word] ) AND (Saudi[Text Word] OR Saudi Arabia[MeSH Terms]) | 1 |
|  | Embase | ('consumer health informatics'/exp OR 'consumer health informatics') AND ('Saudi Arabia'/exp OR 'Saudi') | 1 |
|  | Web of Science | "consumer health informatics" AND (Saudi OR "Saudi Arabia") | 1 |
| Public health informatics (including population informatics and global health informatics) | PubMed | (public health informatics[MeSH Terms] OR public health informatics[Text Word] OR Population informatics[Text Word] OR Global health informatics[Text Word]) AND (Saudi[Text Word] OR Saudi Arabia[MeSH Terms]) | 4 |
|  | Embase | '('public health informatics' OR 'Population informatics' OR 'Global health informatics' ) AND ('Saudi Arabia'/exp OR 'Saudi') | 3 |
|  | Web of Science | ("public health informatics" OR "Population informatics" OR "Global health informatics" ) AND (Saudi OR "Saudi Arabia") | 3 |
| Total |  |  | 1,152 |
