# Supplementary material for: Health informatics publication trends in Saudi Arabia: a bibliometric analysis over the last twenty-four years
Source: J Med Libr Assoc. 2021 Apr 1;109(2):219–39. doi: 10.5195/jmla.2021.1072 (PMC8270356; doi:10.5195/jmla.2021.1072)
Supplement: Supplementary file 2 — Appendix B: Google form for data extraction [file jmla-109-2-219-s02.docx]

# Health informatics publication trends in Saudi Arabia: a bibliometric analysis over the last twenty-four years

## Samar Binkheder; Raniah Aldekhyyel; Jwaher Almulhem

### APPENDIX B

### Google form for data extraction

**Section 1 of 12: Biomedical informatics publication trends**

Abstract ID:

**Section 2 of 12: Publication information**

What is the publication type?

- Research and applications
- Review
- Case reports
- Perspective
- Report
- Correspondence
- Chapter
- Commentary
- Brief communication
- Editorial
- Other (specify next)

**Section 3 of 12: Other options**

You answered “Other” for the publication type. Please specify:

**Section 4 of 12: Publication information, continued**

What is the source of publication?

- Journal
- Proceeding
- Book
- Other (specify next)

**Section 5 of 12: Other source of publication**

You answered “Other” for the source of publication, please specify:

**Section 6 of 12: Author’s information**

Is the Saudi-affiliated author the first author?

- Yes (specify next)
- No

What is/are the Saudi institution/s of author/s (if multiple, please list all separated by "|"):

**Section 7 of 12: Study setting (from abstract or full-text article methodology)**

The location study (from methodology):

**Section 8 of 12: If other country (not listed):**

What is the name of the country (if multiple, please list all separated by "|")?

**Section 9 of 12: Source of data**

Did the study use data?

- Yes (specify next)
- No

**Section 10 of 12: Source of data (if answer is yes)**

If yes, the source of data is (if multiple, please list all separated by "|"):

**Section 11 of 12: Methodology**

What is the type of methodology?

- Qualitative
- Mixed
- Quantitative
- Review
- Other

If the type of methodology is other (if multiple, please list all separated by "|"):

**Section 12 of 12: Any comments/notes about the article**

Please add any comments/observations about the article (if any):
