# Supplementary material for: Health informatics publication trends in Saudi Arabia: a bibliometric analysis over the last twenty-four years
Source: J Med Libr Assoc. 2021 Apr 1;109(2):219–39. doi: 10.5195/jmla.2021.1072 (PMC8270356; doi:10.5195/jmla.2021.1072)

# Health informatics publication trends in Saudi Arabia: a bibliometric analysis over the last twenty-four years

## Samar Binkheder; Raniah Aldekhyyel; Jwaher Almulhem

### APPENDIX C

### (a) Top ten Saudi institutions affiliations for all authors, and (b) the geographical distribution of publications in Saudi’s cities based on author affiliation

To generate the figure, the authors extracted Saudi institutions’ names from authors’ affiliations. The bar chart was generated based on the number of publications for each institution. We found the highest institutions were King Saud bin Abdulaziz University for Health Sciences (KSAU-HS) with 105 (43.4%) publications and King Saud University (KSU) with 58 (24.0%) publications. The city of Riyadh showed the highest number of contributing institutions’ publications. Cities names were collected from these Saudi institutions. The Saudi geographical distribution was generated based on the number of publications per city. Top cities from highest to lowest: Riyadh (n=302), Jeddah (n=39), Dammam (n=13), Meccah (n=8), Dhahran (n=4), Najran (n=2), Hail (n=2), Qassim (n=2), Abha (n=2), Medina (n=2), Khamis Mushait (n=2), Tabuk (n=1), Al-Hada (n=1), Al-Kharj Shaqra (n=1), Taif (n=1), Bisha (n=1), Alhasah (n=1), Jazan (n=1), Jubail City (n=1).

(a) Top 20 Saudi institutions for all authors


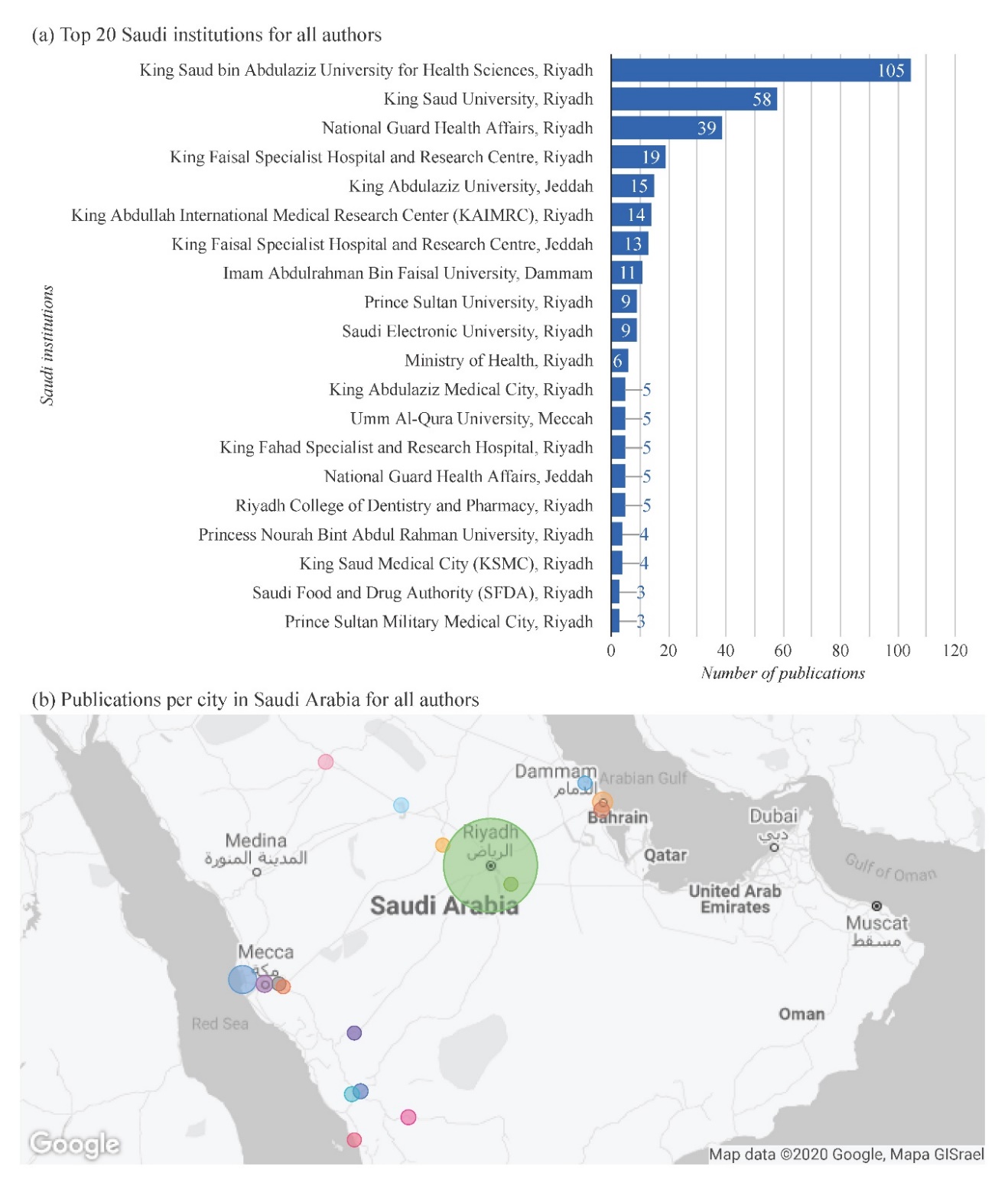


(b) Publications per city in Saudi Arabia for all authors


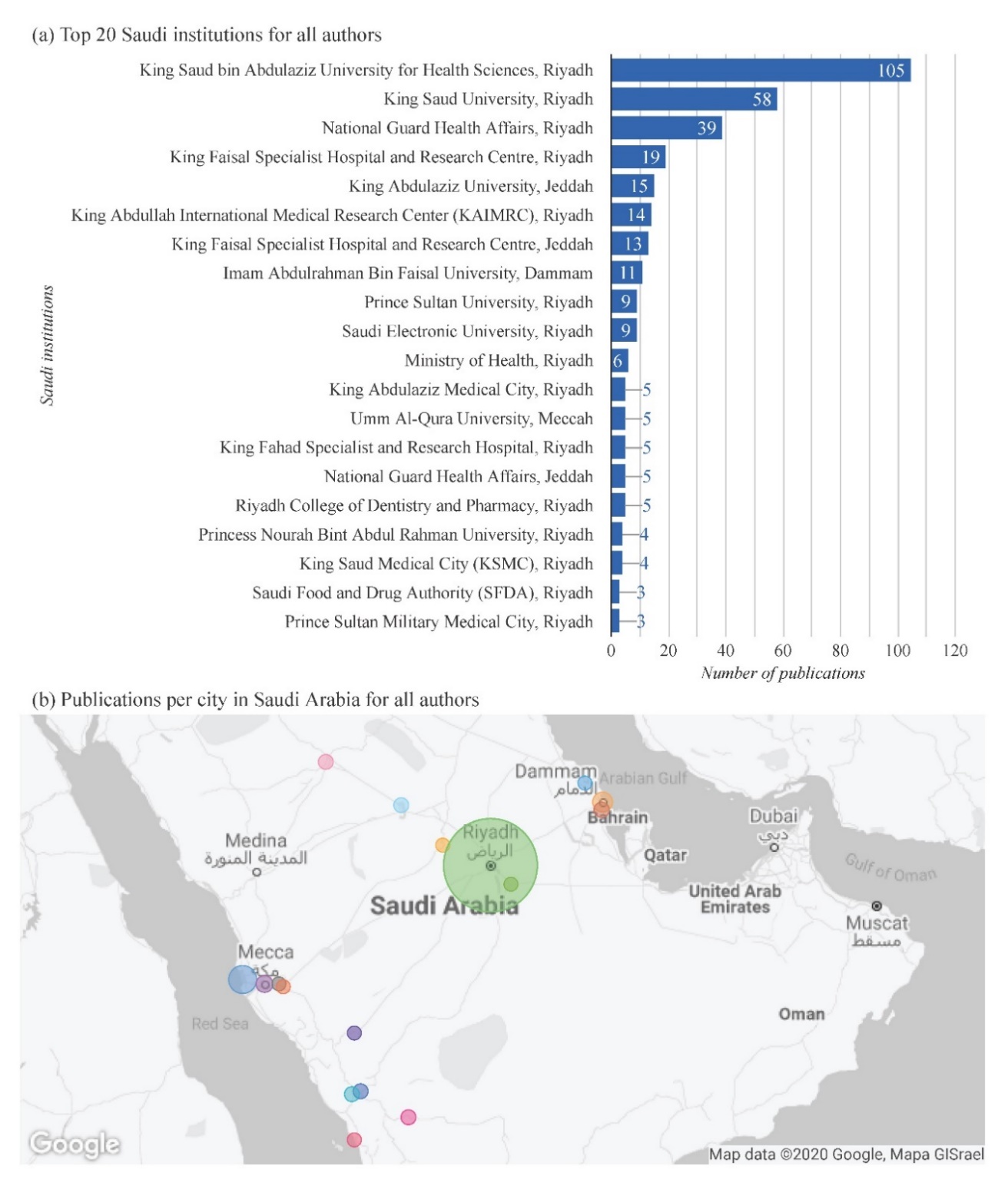

Supplement: Supplementary file 3 — Appendix C: (a) Top ten Saudi institutions affiliations for all authors, and (b) the geographical distribution of publications in Saudi's cities based on author affiliation [file jmla-109-2-219-s03.docx]
